# Impaired aerobic capacity and premature fatigue preceding muscle weakness in the skeletal muscle *Tfam-*knockout mouse model

**DOI:** 10.1242/dmm.048981

**Published:** 2021-09-15

**Authors:** Benjamin Chatel, Sylvie Ducreux, Zeina Harhous, Nadia Bendridi, Isabelle Varlet, Augustin C. Ogier, Monique Bernard, Julien Gondin, Jennifer Rieusset, Håkan Westerblad, David Bendahan, Charlotte Gineste

**Affiliations:** 1 Aix-Marseille Université, CRMBM UMR CNRS 7339, 13385 Marseille, France; 2 CellMade, 73370 Le-Bourget-du-Lac, France; 3 CarMeN Laboratory, UMR INSERM U1060/INRA U1397, Université Claude Bernard Lyon1, F-69310 Pierre-Bénite et F-69500 Bron, France; 4 CarMeN Laboratory, INSERM, INRA, INSA Lyon, Université Claude Bernard Lyon 1, 69600 Oullins, France; 5 Aix-Marseille Université, Université de Toulon, CNRS, LIS, 13397 Marseille, France; 6 Institut NeuroMyoGène, UMR CNRS 5310 - INSERM U1217, Université Claude Bernard Lyon 1, F-69008 Lyon, France; 7 Department of Physiology and Pharmacology, Karolinska Institutet, 17177 Stockholm, Sweden

**Keywords:** Mitochondrial myopathy, Energy metabolism, Muscle fatigue, Exercise intolerance, Muscle weakness

## Abstract

Mitochondrial diseases are genetic disorders that lead to impaired mitochondrial function, resulting in exercise intolerance and muscle weakness. In patients, muscle fatigue due to defects in mitochondrial oxidative capacities commonly precedes muscle weakness. In mice, deletion of the fast-twitch skeletal muscle-specific *Tfam* gene (*Tfam* KO) leads to a deficit in respiratory chain activity, severe muscle weakness and early death. Here, we performed a time-course study of mitochondrial and muscular dysfunctions in 11- and 14-week-old *Tfam* KO mice, i.e. before and when mice are about to enter the terminal stage, respectively. Although force in the unfatigued state was reduced in *Tfam* KO mice compared to control littermates (wild type) only at 14 weeks, during repeated submaximal contractions fatigue was faster at both ages. During fatiguing stimulation, total phosphocreatine breakdown was larger in *Tfam* KO muscle than in wild-type muscle at both ages, whereas phosphocreatine consumption was faster only at 14 weeks. In conclusion, the *Tfam* KO mouse model represents a reliable model of lethal mitochondrial myopathy in which impaired mitochondrial energy production and premature fatigue occur before muscle weakness and early death.

## Abstract (precis)

**Summary:** A time-course study of mitochondrial and muscular dysfunctions in a mouse model of mitochondrial myopathy reveals that decreased resistance to fatigue together with decreased oxidative capacities arise ahead of muscle weakness.

## INTRODUCTION

Mitochondrial diseases are caused by genetic mutations in mitochondrial or nuclear DNA that affect the function of the mitochondrial respiratory chain, thereby leading to defective oxidative phosphorylation (Ishikawa and Nakada, 2021; Parikh et al., 2015; Thorburn, 2004). Skeletal muscles are commonly affected as they require a high amount of energy and are highly dependent on oxidative phosphorylation for energy production. Clinical manifestations of mitochondrial myopathy generally include exercise intolerance, muscle weakness and muscle wasting (Petty et al., 1986). A main histological hallmark of mitochondrial myopathies is the presence of ragged-red fibers, which are muscle fibers exhibiting mitochondrial aggregates in subsarcolemmal regions (Milone and Wong, 2013).

The mitochondrial oxidative phosphorylation system consists of five multi-heteromeric complexes embedded in the inner mitochondrial membrane and composed of subunits encoded by both nuclear and mitochondrial DNA (except complex II, which is only encoded by nuclear DNA) (Larsson and Oldfors, 2001; Rotig and Munnich, 2003). Mitochondrial transcription factor A (Tfam), encoded by nuclear DNA, is a protein with a key role in mitochondrial DNA maintenance (Larsson et al., 1998). Disruption of *Tfam* has been shown to cause a global deficiency of all mitochondrial DNA-encoded proteins, hence affecting complexes I, III, IV and V (Wredenberg et al., 2002).

The mouse model with a selective *Tfam* knockout in fast-twitch skeletal muscle fibers (*Tfam* KO) has been shown to mimic pathological features typically reported in patients with mitochondrial myopathies, including ragged-red fibers with abnormally positioned and enlarged mitochondria, atrophic fibers, COX-deficient fibers and a reduction of respiratory chain enzyme activities (Aydin et al., 2009; Wredenberg et al., 2002). The *Tfam* KO mice do not live more than ∼20 weeks and appear healthy until 12-14 weeks, at which point a progressive clinical phenotype becomes evident (Gineste et al., 2015; Wredenberg et al., 2002). Experiments conducted in isolated whole muscles and intact single muscle fibers of *Tfam* KO mice aged 12-14 weeks have shown muscle weakness (i.e. lower muscle force production) associated with a prolonged and excessive mitochondrial calcium (Ca^2+^) accumulation (Aydin et al., 2009; Gineste et al., 2015). However, muscle fatigue (i.e. a drop in force during sustained or repeated contractions) remained unaffected and has been explained by an increased proportion of the muscle fiber volume being occupied by mitochondria combined with a lower energy expenditure during contractions. This reduced cost would be due to a reduced sarcoplasmic reticulum (SR) calcium (Ca^2+^) release resulting in a decreased activation of the energy-consuming cross-bridges and SR Ca^2+^ pumps (Aydin et al., 2009; Wredenberg et al., 2002). A similar lack of effect on muscle fatigue has been previously demonstrated in nemaline myopathy mouse models that also display severe muscle weakness (Gineste et al., 2013, 2020); therefore, this lack of effect may also be due to the fact that experiments have been conducted in relatively late stages of the disease, i.e. when severe muscle weakness was established and the resulting energy consumption during contractions was decreased. Accordingly, premature fatigue (i.e. a faster drop in force) related to decreased mitochondrial oxidative capacity has been shown to precede overt muscle weakness in mouse models with mitochondrial dysfunction, as well as in patients with mitochondrial myopathies (Finsterer, 2020; Tarnopolsky, 2016; Yamada et al., 2012).

In the present study, we hypothesized that faster muscle fatigue and impaired mitochondrial energy production precede muscle weakness in the *Tfam* KO mouse model. *In vivo* metabolic changes, force production and muscle fatigue were assessed in *Tfam* KO mice at two different timepoints, i.e. before mice enter the stage with obvious symptoms (11 weeks old) and when mice are about to enter the terminal stage with progressive muscle weakness (14 weeks old). These *in vivo* investigations of the skeletal muscle function were combined with *in vitro* experiments on isolated muscles to study whether there were structural alterations indicating increased mitochondrial Ca^2+^ influx via mitochondria-associated membranes (MAM) (Csordas et al., 2018), which might explain the excessive mitochondrial Ca^2+^ uptake observed in *Tfam* KO muscle fibers during repeated contractions (Aydin et al., 2009; Gineste et al., 2015).

## RESULTS

### Progressive bodyweight loss is associated with skeletal muscle atrophy without fatty replacement of muscle in *Tfam* KO mice

Skeletal muscle atrophy and replacement of skeletal muscle tissue by fat deposition or fibrotic tissue is a frequent feature in muscular disorders and has been reported in patients with mitochondrial myopathy, although it is not a common feature (Olsen et al., 2003; Theodorou et al., 2012). Therefore, we measured bodyweight and the volume of hindlimb muscles using magnetic resonance imaging (MRI) in *Tfam* KO mice and their control littermates (wild type). We also determined whether muscle was replaced by fatty infiltration.

**Fig. 1.**
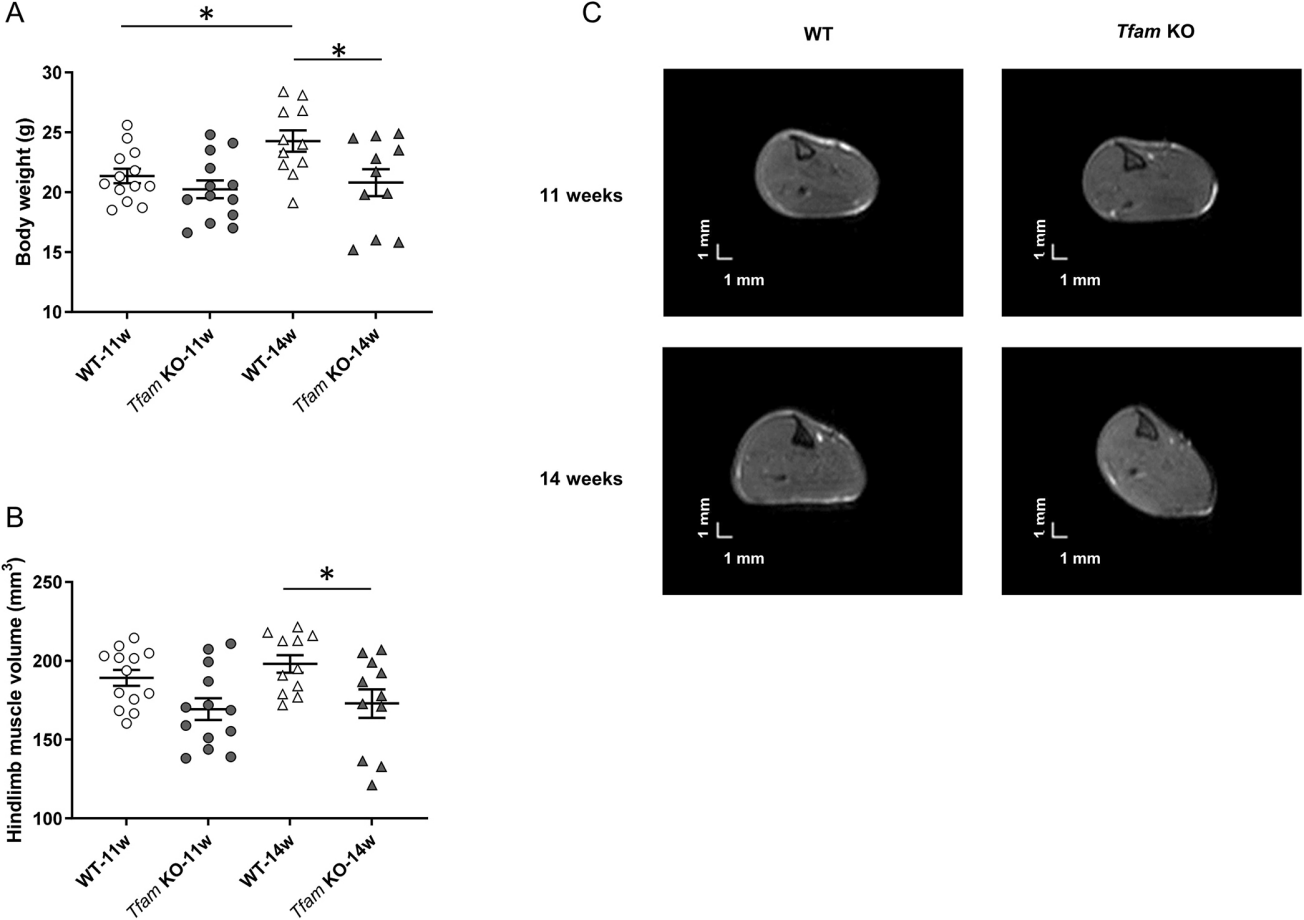
***Tfam* KO mice exhibit reduced bodyweight associated with muscle atrophy at 14 weeks.** (A) Body mass in wild-type (WT) and *Tfam* KO mice at 11 and 14 weeks of age. (B) Volume of hindlimb muscles in wild-type and *Tfam* KO mice. WT-11w, *n*=13; *Tfam* KO-11w, *n*=13; WT-14w, *n*=11; *Tfam* KO-14w, *n*=11. Data presented as individual values and are mean±s.e.m. *P<0.05 (two-way ANOVA and Sidak's post-hoc test). (C) Representative axial magnetic resonance images obtained from wild-type (left panels) and *Tfam* KO (right panels) at 11 (top panels) and 14 weeks (bottom panels) of age.

At 11 weeks of age, *Tfam* KO and wild-type mice had a similar bodyweight, whereas at 14 weeks bodyweight was ∼15% lower in *Tfam* KO than in wild-type mice (Fig. 1A). Although no difference was observed between *Tfam* KO mice between the two ages, bodyweight was higher in wild-type mice at 14 weeks compared to 11 weeks.

The volume of hindlimb muscles was ∼15% lower in *Tfam* KO than in wild-type mice at 14 weeks, whereas there was no difference at 11 weeks (Fig. 1B,C), indicating muscle atrophy at 14 weeks in *Tfam* KO mice. No difference was observed between 11 weeks and 14 weeks for the two genotypes. Neither intramuscular fatty infiltration nor fibrotic tissue was detected in *Tfam* KO mice at either age, as illustrated by the absence of pixels with higher intensities in the muscle region (Fig. 1C).

### Reduced maximal force at 14 weeks in *Tfam* KO mice is related to muscle atrophy

Patients with mitochondrial myopathies usually present skeletal muscle weakness (i.e. decreased force production) (Moggio et al., 2014), which is a common feature reported in neuromuscular diseases (Ahmed et al., 2018; Scelsi, 1992) and may be due to skeletal muscle atrophy and/or impaired contractile function within the muscles. To determine whether the *Tfam* KO mice showed skeletal muscle weakness, we measured maximal force production (at 150 Hz) and submaximal force production (at 20 Hz, which is <50% of maximal force) in the unfatigued state. We assessed both absolute force and specific force, i.e. force normalized to hindlimb muscle volume to assess contractile function independently of hindlimb muscle size.

**Fig. 2.**
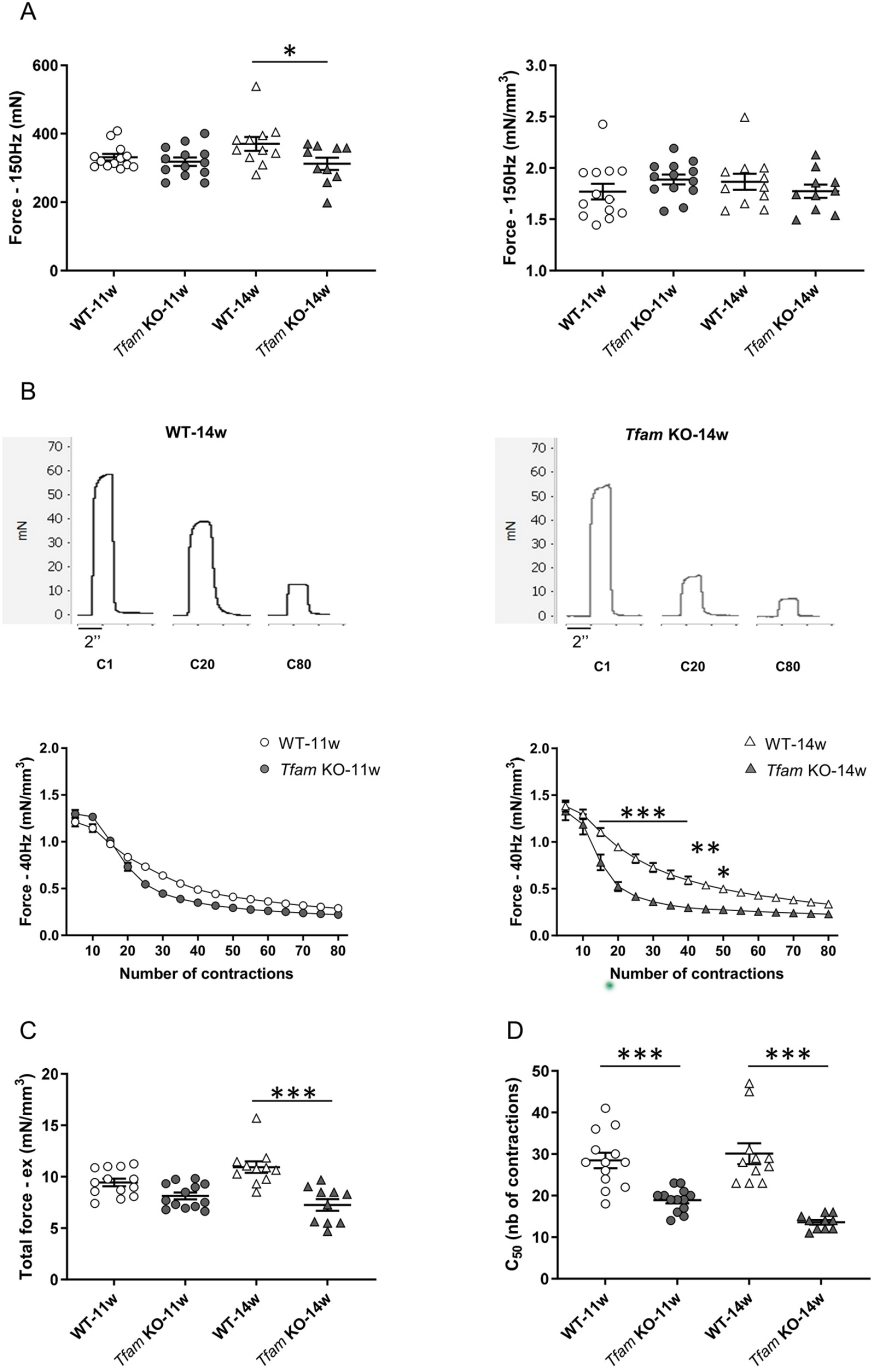
***Tfam* deletion resulted in accelerated muscle fatigue in mice at 11 and 14 weeks of age, and muscle weakness only at 14 weeks of age.** (A) Maximal (150 Hz) absolute (left panel) and specific (right panel) force of the plantar flexor muscles *in vivo*. (B) Representative force traces of the plantar flexor muscles in wild-type (WT) and *Tfam* KO mice at 14 weeks of age, recorded during the first contraction (C1), 20th contraction (C20) and last contraction (C80) of the 80 contractions of fatiguing stimulation (top panels), and average data of *in vivo* specific force (bottom panels). (C) Total specific force of the plantar flexor muscles produced during the whole fatiguing protocol. (D) Contraction at which force production was 50% of the unfatigued force (C_50_). WT-11w, *n*=13; *Tfam* KO-11w, *n*=13; WT-14w, *n*=11; *Tfam* KO-14w, *n*=10. Data presented as individual values and are mean±s.e.m. for panels A and C. Data are mean±s.e.m. for panel B. *P<0.05, **P<0.01, ***0.001. Statistical significance was determined using two-way ANOVA with repeated measures on contraction number, Tukey's post-hoc test for force during exercise, and two-way ANOVA for all other comparisons with Sidak's post-hoc test for absolute force and Tukey's post-hoc test for total force and C_50_. There was no statistically significant difference between all groups for normalized force at 150 Hz when two-way ANOVA was applied.

Absolute maximal tetanic forces did not differ between *Tfam* KO and wild-type mice at 11 weeks, whereas force was ∼15% lower in *Tfam* KO mice at 14 weeks (Fig. 2A). When normalized to the volume of hindlimb muscles, force was similar in *Tfam* KO and wild-type mice at both ages, indicating that muscle weakness at 14 weeks in *Tfam* KO mice is related to muscle atrophy. No difference in maximal force was observed between 11 weeks and 14 weeks for the two genotypes. No significant difference was found for absolute and specific submaximal forces between groups for the same age. Nevertheless, both absolute and specific force at 20 Hz increased for wild-type mice between 11 and 14 weeks, whereas no significant change was observed for *Tfam* KO mice (Fig. S1).

### Faster fatigue in *Tfam* KO than in wild-type mice at 11 and 14 weeks of age

Premature muscle fatigue (i.e. faster decline in force during sustained or repeated contractions) is a typical feature in patients with mitochondrial myopathy (Finsterer, 2020; Tarnopolsky, 2016). We measured force production during 80 repeated submaximal contractions (1.5 s contractions given every 7.5 s). Specific force was significantly decreased in *Tfam* KO mice from the 15th to the 50th contraction at 14 weeks, whereas it was similar between the two groups at 11 weeks (Fig. 2B,C). Absolute force was significantly lower in *Tfam* KO than in wild-type mice from the 25th to the 35th contraction at 11 weeks old and from the tenth to the 50th contraction at 14 weeks of age (Fig. S2).

We performed two additional measurements to characterize changes in force production during fatiguing stimulation. First, the total specific force produced throughout the exercise period was similar between wild-type and *Tfam* KO mice at 11 weeks of age, but was significantly reduced by ∼35% at 14 weeks of age in *Tfam* KO mice compared to wild-type mice (Fig. 2C). We also measured the contraction that gives 50% of the initial force (C_50_) because the rate of decline in force differed largely in the initial part of fatiguing stimulation between *Tfam* KO and wild-type mice. The results revealed a markedly faster initial force decline (lower C_50_) in *Tfam* KO than in wild-type mice at both ages (Fig. 2D), indicating that fatigue was faster in *Tfam* KO mice at both ages. It is worth noting that force reached a relatively stable low level towards the end of fatiguing stimulation, during which forces were similar in *Tfam* KO and wild-type mice (see Fig. 2B). One likely explanation for this is that towards the end of the stimulation period, force was maintained primarily by highly fatigue resistant slow-twitch muscle fibers, which are not genetically afflicted in the fast-twitch muscle fiber-specific *Tfam* KO mouse.

### Muscle energetic status at rest is affected in *Tfam* KO mice at both ages

**Fig. 3.**
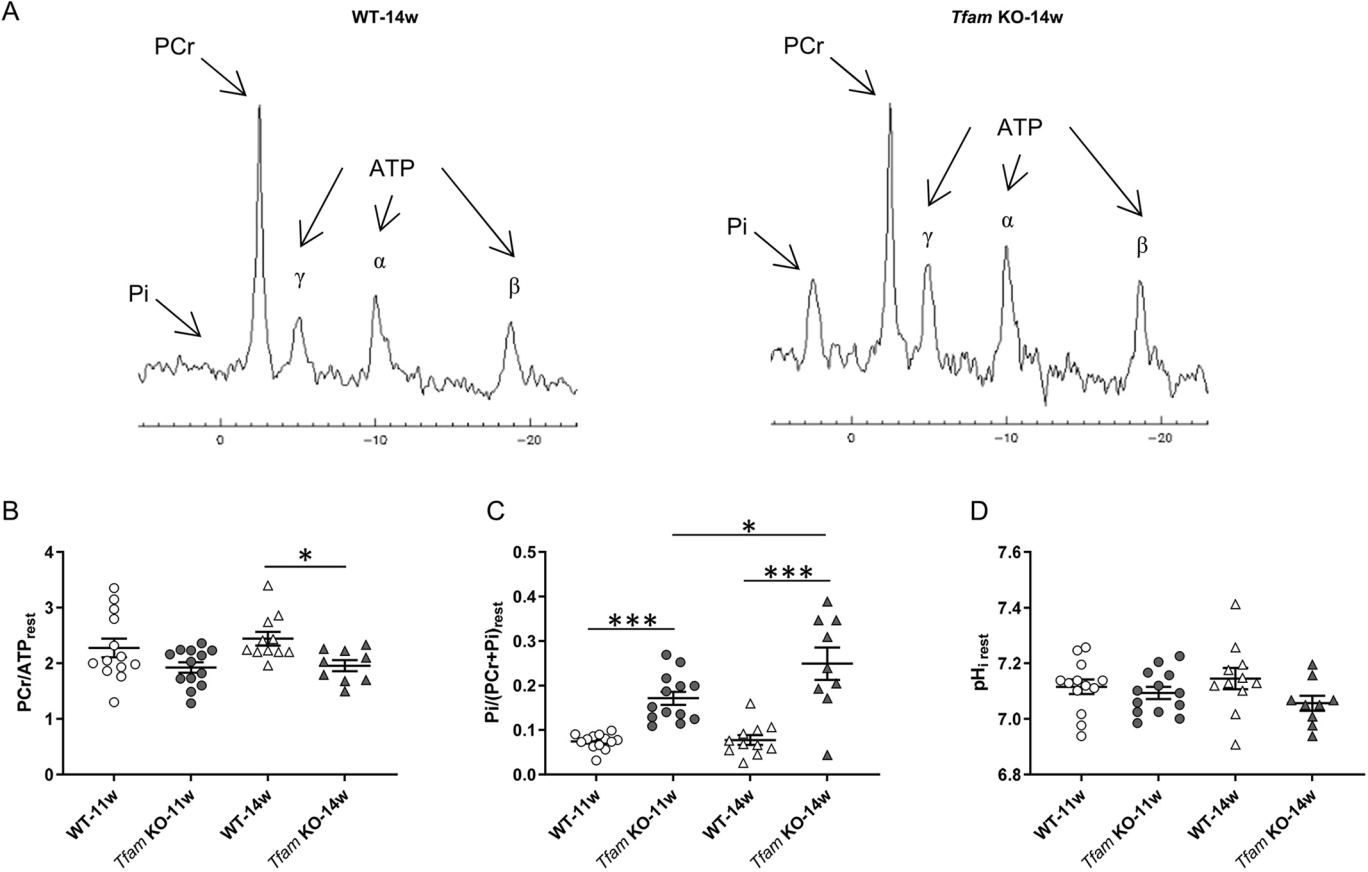
**Metabolic parameters at rest were altered in *Tfam* KO mice and progressed between 11 and 14 weeks of age.** (A) Example of resting ^31^P-MR spectra in wild-type (WT) and *Tfam* KO muscles at 14 weeks of age. (B-D) Phosphocreatine (B), inorganic phosphate (C) and pH_i_ (D) measured at rest. WT-11w, *n*=13; *Tfam* KO-11w, *n*=13; WT-14w, *n*=11; *Tfam* KO-14w, *n*=9. Data presented as individual values and are mean±s.e.m. *P<0.05, ***0.001 (two-way ANOVA with Sidak's post-hoc test for PCr/ATP_rest_ and Tukey's post-hoc test for Pi/(PCr+Pi)_rest_; no significant difference was found for pH_i rest_ with two-way ANOVA). Pi, inorganic phosphate.

Patients with mitochondrial myopathy present impaired muscle metabolism at rest and particularly during exercise (Argov et al., 1987; Matthews et al., 1991; Radda et al., 1985; Taylor et al., 1994). Thus, we used phosphorus-31 magnetic resonance spectroscopy (^31^P-MRS) to study differences in energy metabolites at rest and during repeated contractions (Fig. 3A). At rest, the phosphocreatine to ATP ratio was not significantly different between *Tfam* KO and wild-type mice at 11 weeks of age but was significantly lower at 14 weeks (Fig. 3B). Resting inorganic phosphate was significantly higher in *Tfam* KO than in wild-type mice at both ages, and this metabolic impairment in *Tfam* KO mice progressed between 11 and 14 weeks of age (Fig. 3C). Resting pH_i_ was not significantly different between groups at either 11 or 14 weeks of age (Fig. 3D). The increase in inorganic phosphate together with the reduced phosphocreatine to ATP ratio at 14 weeks of age in *Tfam* KO mice indicate an impaired metabolic regulation with inadequate matching between oxidative ATP supply and demand in the resting state.

### Impaired muscle energy metabolism during fatiguing contractions in *Tfam* KO mice at 11 and 14 weeks of age

**Fig. 4.**
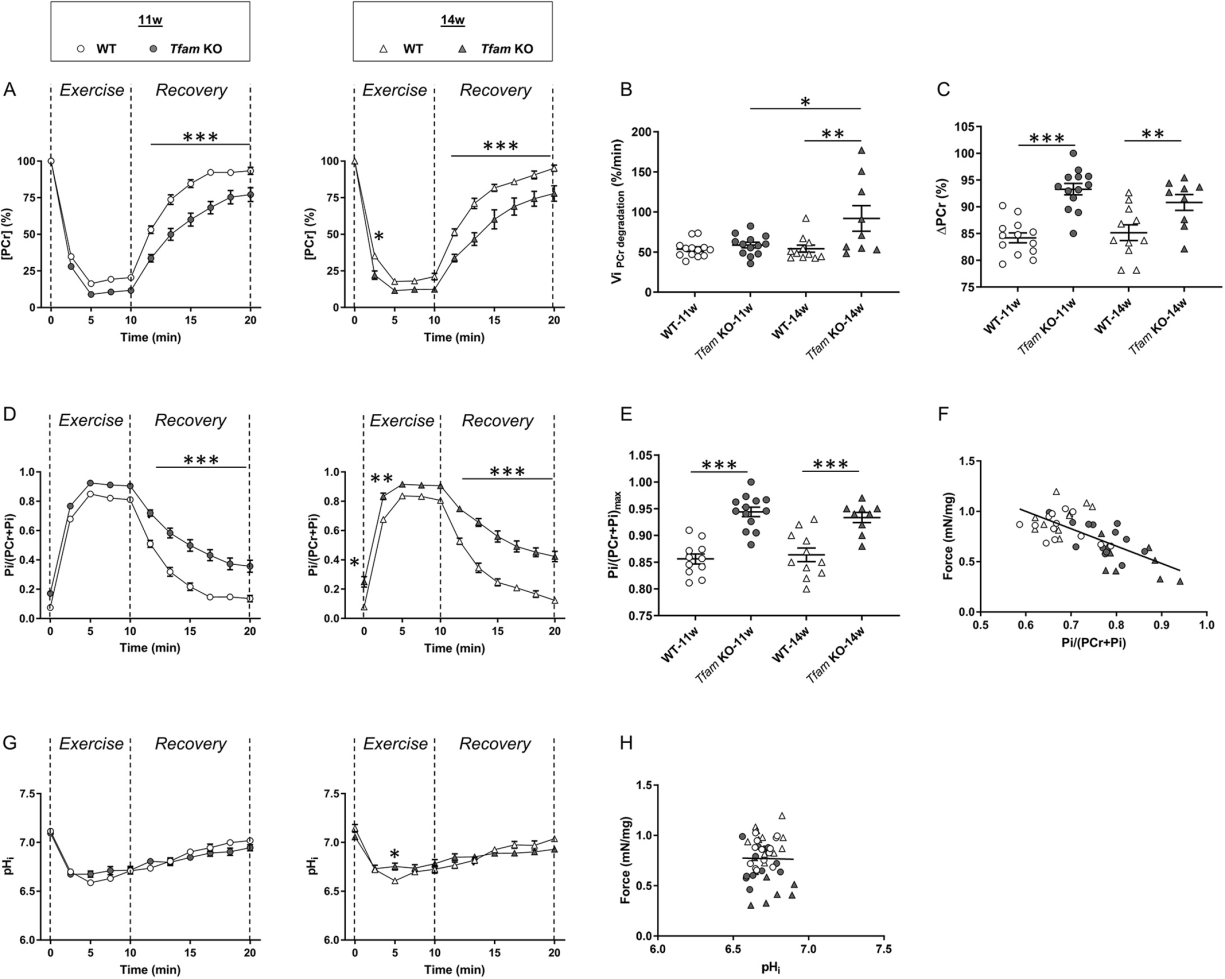
**Impaired phosphocreatine and inorganic phosphate metabolism during exercise *in Tfam* KO mice.** (A) Phosphocreatine levels throughout the stimulation period and during the recovery after the stimulation. (B,C) Initial rate of phosphocreatine degradation (B) and total magnitude of phosphocreatine reduction (C) during the stimulation period. (D) Inorganic phosphate levels during fatiguing stimulation and recovery. (E) Maximal inorganic phosphate reached during fatiguing protocol. (F) Relationship between force and inorganic phosphate after 2.5 min of fatiguing exercise (r=−0.69; *P*<0.001). (G) pH_i_ during stimulation and recovery periods. (H) Relationship between force and pH_i_ after 2.5 min of fatiguing exercise (r=−0.01; *P*=0.93). WT-11w, *n*=13; *Tfam* KO-11w, *n*=13; WT-14w, *n*=11; *Tfam* KO-14w, *n*=9. Data are mean±s.e.m. for panels A, D and G. Data presented as individual values and are mean±s.e.m. for other panels. *P<0.05, **0.01, ***0.001 (two-way ANOVA with repeated measures on time and Tukey's post-hoc test for time course of phosphocreatine, inorganic phosphate and pH_i_; two-way ANOVA for all other comparisons except linear regressions with Sidak's post-hoc test for ΔPCr and Pi/(PCr+Pi)_max_, and Tukey's post-hoc test for Vi_PCr degradation_). Pi, inorganic phosphate; Vi_PCr degradation_, initial rate of phosphocreatine breakdown.

We also investigated muscle bioenergetics during a standardized fatiguing exercise protocol through the time-course changes in phosphocreatine, inorganic phosphate and pH_i_. During contractile activity, skeletal muscles require ATP produced by both anaerobic and oxidative metabolism. The major anaerobic ATP sources are breakdown of phosphocreatine, which results in the accumulation of inorganic phosphate ions, and glycolysis ending with lactate and hydrogen ion (H^+^) formation. During the first 2 min of exercise, phosphocreatine decreased rapidly and then reached a stable low level at 5 min of stimulation (Fig. 4A). The initial rate of phosphocreatine consumption was similar in *Tfam* KO and wild-type mice at 11 weeks of age, whereas it was ∼70% higher in *Tfam* KO mice than in wild-type mice at 14 weeks of age (Fig. 4B). The magnitude of phosphocreatine depletion was significantly larger in the *Tfam* KO mice than in wild-type mice at both 11 and 14 weeks of age (Fig. 4C). The increase in inorganic phosphate evolved as a mirror of phosphocreatine, i.e. inorganic phosphate levels increased rapidly during the first 2 min of exercise followed by a plateau from 5 min to the end of the stimulation period (Fig. 4D,E). Increased cytosolic inorganic phosphate levels are considered to be a major cause of force decline in skeletal muscle fatigue (Westerblad and Allen, 1996). With this in mind, we plotted the relationship between force and inorganic phosphate after 2.5 min of fatiguing stimulation (i.e. covering the part of fatiguing stimulation at which both these parameters showed rapid changes) for individual mice at 11 and 14 weeks of age (Fig. 4F), and found a strong significant negative correlation between these two variables (r=−0.69; *P*<0.001).

The decrease in pH_i_ during the fatiguing stimulation was similar in *Tfam* KO and wild-type mice, with the only exception being significantly less acidosis at 5 min of stimulation in *Tfam* KO compared to wild-type mice at 14 weeks of age (Fig. 4G). Decreased pH_i_ is commonly considered to be a major cause of force decline in fatigued skeletal muscle, although its role is currently debated (Westerblad, 2016). Plotting of the relationship between force and pH_i_ after 2 min of fatiguing stimulation did not reveal any significant correlation (r=−0.01; *P*=0.93; Fig. 4H).

### Slowed recovery kinetics of phosphocreatine in *Tfam* KO mice at 11 and 14 weeks of age

**Fig. 5.**
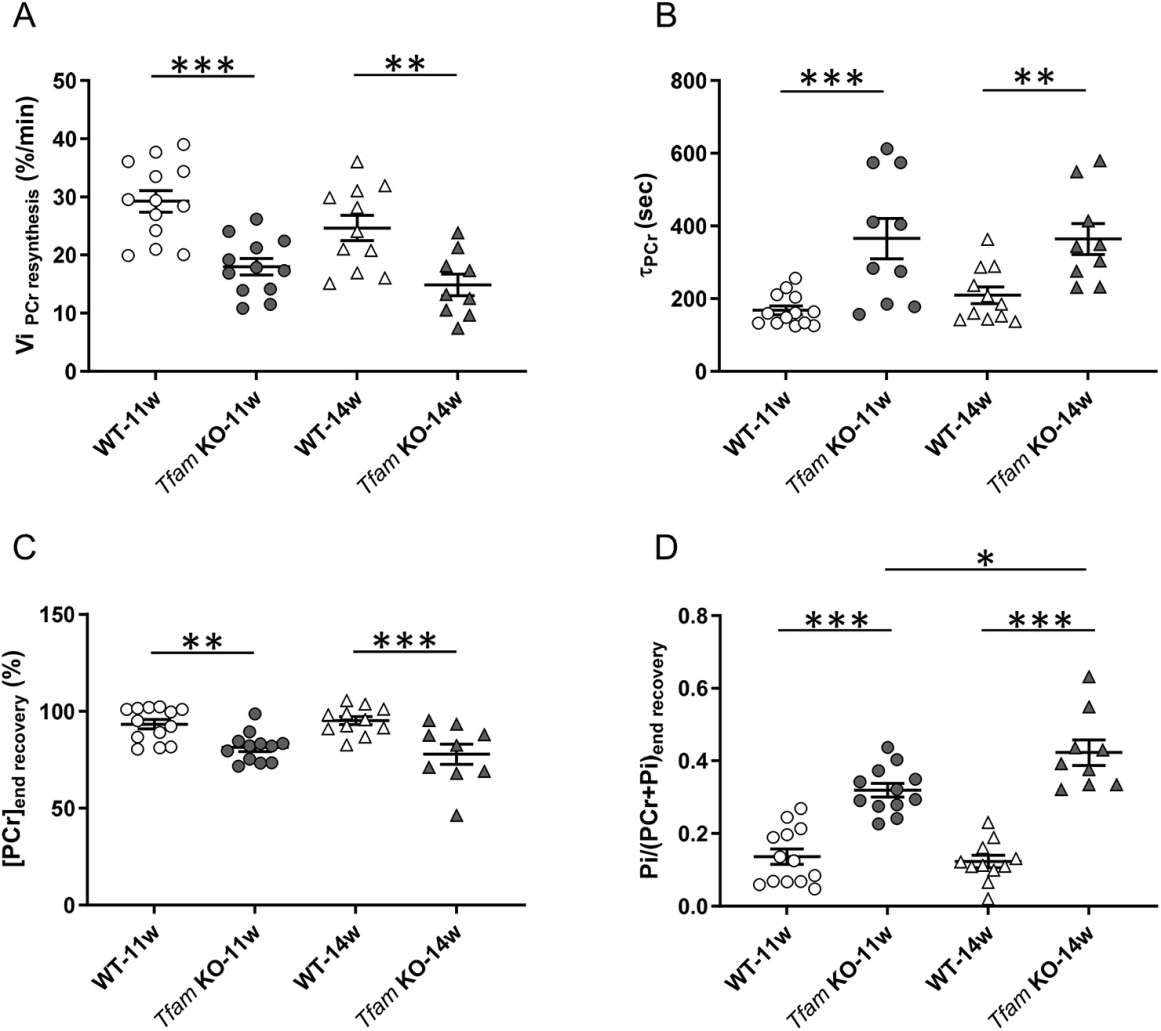
**Slowed recovery of metabolites implies decreased mitochondrial oxidative capacity in 11- and 14-week-old *Tfam* KO mice.** (A) Initial rate of phosphocreatine resynthesis after the stimulation bout. (B) Time constant of phosphocreatine recovery during the 10 min recovery phase after exercise. (C) Phosphocreatine at the end of the recovery period. (D) Inorganic phosphate at the end of the 10 min recovery period. WT-11w, *n*=13; *Tfam* KO-11w, *n*=12 for all analyses except for T_Phosphocreatine_ for which *n*=10; WT-14w, *n*=11; *Tfam* KO-14w, *n*=9. Data presented as individual values and are mean±s.e.m. *P<0.05, **0.01, ***0.001 (two-way ANOVA with Tukey's post-hoc test for Pi/(PCr+Pi)_end recovery_ and Sidak's post-hoc test for all other comparisons). Pi, inorganic phosphate; Vi_PCr resynthesis_, initial rate of phosphocreatine resynthesis.

Phosphocreatine recovery kinetics are related to the end-exercise rate of oxidative ATP synthesis and a slowed phosphocreatine recovery has been recognized as indicative of an impaired mitochondrial oxidative function (Arnold et al., 1984), and a slower rate has been commonly reported in patients with mitochondrial myopathies (Barbiroli et al., 1997; Bendahan et al., 1992; Taylor et al., 1994). We evaluated the recovery time courses of both phosphocreatine and inorganic phosphate for 10 min after the end of the fatiguing protocol (see Fig. 4A,B). The initial rate of phosphocreatine recovery was significantly lower in *Tfam* KO than in wild-type mice at both 11 and 14 weeks of age (Fig. 5A). Likewise, at both ages, the time constant of phosphocreatine recovery (T_PCr_) was significantly longer in the *Tfam* KO than in wild-type mice (Fig. 5B), and phosphocreatine was still ∼15% lower in *Tfam* KO than in wild-type mice at the end of the 10-min recovery period (Fig. 5C; Table S1). Additionally, phosphocreatine was not fully returned to resting levels after the 10 min of recovery in *Tfam* KO mice (Fig. 4A; Table S1). The decrease in inorganic phosphate during recovery followed the same time course and was significantly higher in *Tfam* KO than in wild-type mice at the end of the recovery period at both ages (Fig. 5D). Moreover, inorganic phosphate at the end of the recovery period was significantly higher than the resting level in *Tfam* KO mice (Fig. 4D; Table S1). During the post-stimulation recovery period, pH_i_ gradually increased towards its resting level at a similar rate in *Tfam* KO and wild-type mice (Fig. 4G).

### The contact points at the mitochondria-associated membrane level are decreased in *Tfam* KO muscle

**Fig. 6.**
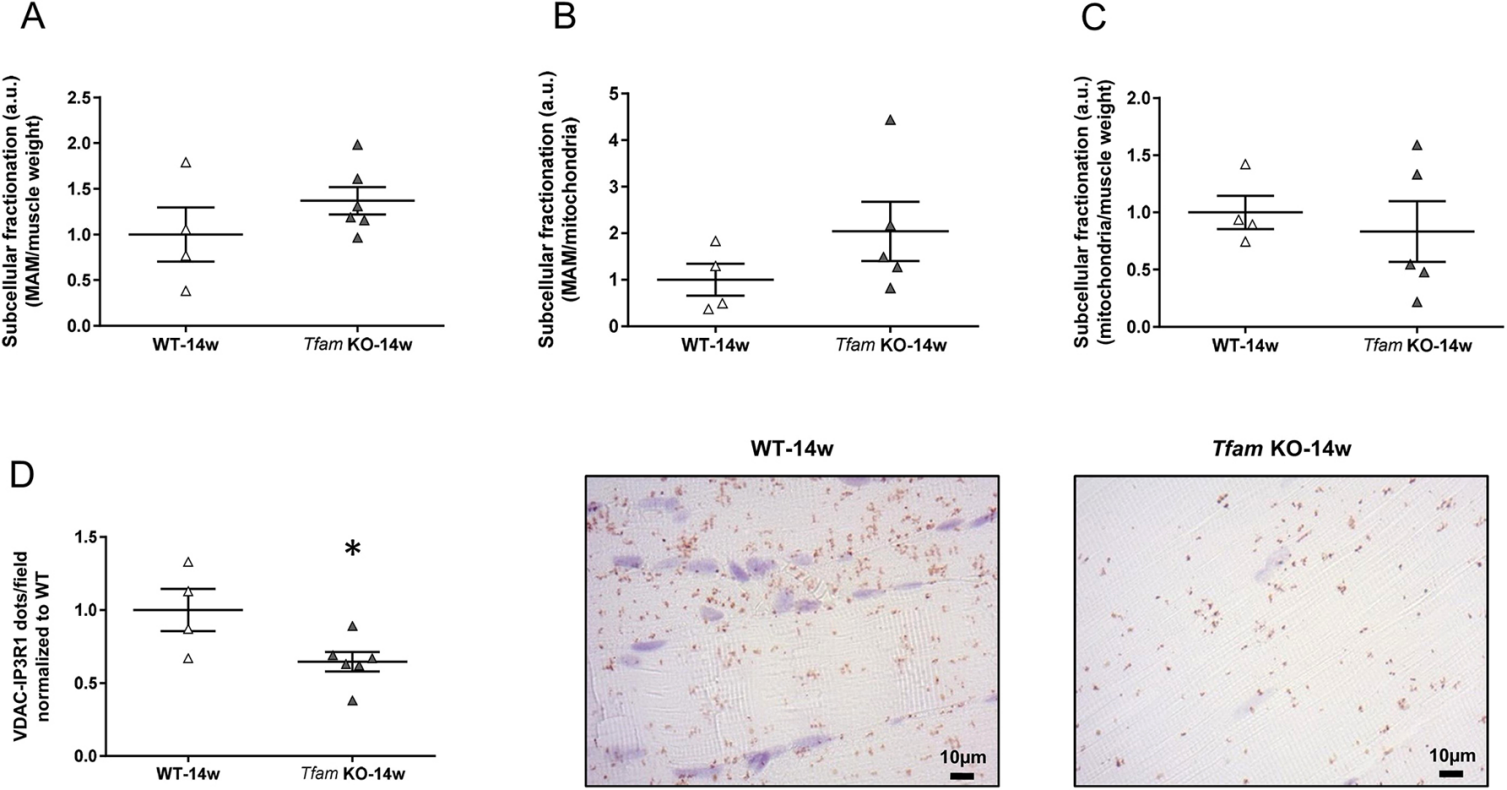
***Tfam* KO mice show decreased interaction points between the sarcoplasmic reticulum and mitochondria.** (A,B) Quantification of the normalized protein ratio of MAM over muscle weight (A) and over pure mitochondria (B) from gastrocnemius muscle fractionation. (C) Quantification of the normalized protein ratio of mitochondria over muscle weight from gastrocnemius muscle fractionation in *Tfam* KO and wild-type (WT) mice. (D) Quantitative analysis of VDAC1-IP3R1 interactions (left panel) and representative images of the VDAC1-IP3R1 interactions (brown dots) measured by *in situ* PLA in paraffin-embedded gastrocnemius muscle in wild-type (top-right panel) and *Tfam* KO mice (bottom-right panel). Data presented as individual values and are mean±s.e.m. and normalized to the mean value in wild-type muscles, which was set to 1.0. *P<0.05 (unpaired Mann–Whitney test for MAM; two-tailed unpaired *t*-test for VDAC-IP3R1). a.u., arbitrary units.

To study whether alterations in MAM might be involved in the excessive mitochondrial Ca^2+^ uptake in *Tfam* KO muscle fibers (Aydin et al., 2009; Gineste et al., 2015), mitochondria and MAM were isolated from gastrocnemius muscles of 14-week-old wild-type and *Tfam* KO mice. No statistically significant difference between wild-type and *Tfam* KO mice was observed for the MAM protein content related to muscle weight (Fig. 6A) or isolated mitochondria (Fig. 6B), or for total mitochondrial protein content related to muscle weight (Fig. 6C). The physical SR-mitochondria interaction, assessed as the *in situ* protein-protein interaction between the SR Ca^2+^ channel inositol 1,3,4-triphosphate receptor 1 (IP_3_R1, also known as ITPR1) and the mitochondrial outer membrane voltage-dependent anion channel 1 (VDAC1) (Szabadkai et al., 2006), was significantly lower in *Tfam* KO than in wild-type mice (Fig. 6D).

## DISCUSSION

In this study, we used a mouse model of lethal mitochondrial myopathy, the fast-twitch skeletal muscle-specific *Tfam* KO mouse. Experiments were performed at an early state of the disease process and our results show impaired mitochondrial energy production accompanied by a faster force decline during repeated contractions in *Tfam* KO than in wild-type mice; these impairments occurred before any signs of reduced hindlimb muscle volume and decreased force production in the unfatigued state. In other words, muscles of *Tfam* KO mice showed an increased muscle fatigue before entering the state with progressive muscle atrophy and muscle weakness. These results extend those from previous studies showing severe muscle weakness in more advanced stages of the disease (Gineste et al., 2015; Wredenberg et al., 2002), and support those reported in patients related to a progression from early premature fatigue to disabling muscle weakness at rest (Finsterer, 2020; Tarnopolsky, 2016).

Contractile function in the unfatigued state was impaired in *Tfam* KO mice only at 14 weeks of age and at maximal force, and was directly related to the skeletal muscle atrophy occurring at this age as normalized force was not different from that in wild-type mice. The absence of muscle weakness independent from muscle atrophy at maximal and submaximal forces contrasts with previous results showing a ∼20% reduction in the force produced by isolated single fast-twitch muscle fibers of *Tfam* KO mice tested at a similar stage of the disease process, which was related to decreased SR Ca^2+^ release (Aydin et al., 2009). This apparent difference might be due to the fact that, *in vivo*, decreased muscle fiber force production might be compensated by various non-contractile structures contributing to force transmission, which are partly missing in experiments on isolated single fibers (Cornwall, 1984; Ward et al., 2020). A decreased force production in fast-twitch muscle fibers of *Tfam* KO mice might also be masked in the present *in vivo* experiments by a substantial proportion of the muscle being occupied by normally contracting slow-twitch fibers in which *Tfam* was not knocked out (Wredenberg et al., 2002). In this context, it can be noted that the maintained low force levels observed towards the end of fatiguing stimulation likely reflect sustained force production in fatigue-resistant slow-twitch fibers. Accordingly, these maintained low force levels were of similar size in *Tfam* KO and wild-type mice, which fits with the normal genetic status of slow-twitch fibers in our *Tfam* KO mouse model.

Conversely, muscle fatigue induced by repeated submaximal contractions developed markedly faster at both 11 and 14 weeks of age in *Tfam* KO mice compared to wild-type mice, but was associated with reduced force production in the unfatigued state only at 14 weeks of age. This result highlights that accelerated muscle fatigue precedes muscle weakness in *Tfam* KO mice. The altered muscle fatigue was particularly marked in the initial part of fatiguing stimulation. This early undue fatigue in the *Tfam* KO mice clearly mirrors the exercise intolerance commonly observed in patients with mitochondrial myopathy (Taivassalo and Haller, 2005; Taivassalo et al., 2003). Accelerated fatigue during repeated tetanic stimulation was not previously reported in single muscle fibers isolated from *Tfam* KO mice at a similar stage in the disease process (Aydin et al., 2009). Three differences between the present and the previous study might explain this discrepancy. First, tetanic force and free cytosolic [Ca^2+^] ([Ca^2+^]_i_) were already reduced at the start of fatiguing stimulation in the previous single fiber experiments. This means that less energy was used for crossbridge cycling and SR Ca^2+^ reuptake during contractions, and hence the stress on mitochondrial energy production was lowered, which would slow fatigue development (Cheng et al., 2019). Second, fatigue was induced by repeated submaximal (∼50% of the maximal force) contractions in the present study, whereas close to maximal (∼85% of the maximal force) contractions were used in the previous single fiber study. Submaximal contractions are on the steep part of the force-[Ca^2+^]_i_ relationship where fatigue-induced decreases in tetanic [Ca^2+^]_i_ and myofibrillar Ca^2+^ sensitivity have a much larger impact than with near-maximal contractions (Allen et al., 2008; Place et al., 2010). Third, the knockout of *Tfam* in fast-twitch skeletal muscle fibers might cause additional maladaptive modifications that limit *in vivo* fatigue but have no direct impact on the fatigue of isolated muscle fibers. For instance, slower heart rate kinetics at the onset of exercise have been observed in mitochondrial myopathy patients (Grassi et al., 2009).

Our results show clear-cut signs of impaired intracellular energy metabolism before, during and after the fatiguing stimulation bout in *Tfam* KO muscles at 11 and 14 weeks of age, which is consistent with the impaired mitochondrial oxidative metabolism observed in mitochondrial myopathy patients (Arnold et al., 1985; Hayes et al., 1985; Matthews et al., 1991; Moller et al., 2005; Taylor et al., 1994). In the resting state, *Tfam* KO muscles displayed a progressive increase in inorganic phosphate, which would be required to maintain an adequate rate of mitochondrial ATP synthesis as mitochondrial function declines (Wu et al., 2007).

During the fatiguing protocol, phosphocreatine breakdown was larger in *Tfam* KO mice at both ages and faster in the initial part of the exercise at 14 weeks of age. These changes in phosphocreatine were mirrored by an increase in inorganic phosphate and indicate an increased dependency on phosphocreatine hydrolysis to maintain ATP levels during repeated contractions. Moreover, we observed a good correlation between the increase in inorganic phosphate and the decrease in force during the stimulation period (see Fig. 4F), which fits with inorganic phosphate being a major causative factor in acute muscle fatigue that afflicts crossbridge force production, myofibrillar Ca^2+^ sensitivity and SR Ca^2+^ release (Allen et al., 2008).

In addition to phosphocreatine breakdown, the major anaerobic energy source is glycolysis ending with the formation of lactate and H^+^ when coupled to ATP hydrolysis. Intramuscular acidosis has not been reported as a distinctive characteristic of mitochondrial diseases (Arnold et al., 1985; Hayes et al., 1985; Taylor et al., 1994). Accordingly, resting pH_i_ was unaltered in *Tfam* KO mice at both ages, in agreement with reports from patients with mitochondrial myopathies (Arnold et al., 1985; Hayes et al., 1985; Moller et al., 2005). Moreover, we observed no difference in pH_i_ changes during exercise and recovery periods between *Tfam* KO and wild-type mice, except for a slightly larger acidification after 5 min of stimulation in wild-type mice at 14 weeks of age. These results imply that the faster fatigue in *Tfam* KO muscle is independent of acidosis. This implication is emphasized by the lack of correlation between decreased pH_i_ and reduced force during fatiguing stimulation (see Fig. 4H), and fits with previous results demonstrating that acidosis is not the primary cause of exercise intolerance in patients with mitochondrial myopathy (Vissing et al., 2001).

Finally, a larger PCr consumption together with a similar or a lower force production (11 and 14 weeks of age, respectively) might reflect a higher energy cost of contraction. It can be noted that during the first minute of fatiguing stimulation, when ATP production relies on anaerobic processes in both *Tfam* KO and wild-type muscles (Lannergren and Westerblad, 1991), the rate of force decline was similar in *Tfam* KO and wild-type muscles (see Fig. 4B). Thereafter, when aerobic ATP production becomes increasingly more important, fatigue developed at a faster rate in *Tfam* KO than in wild-type muscles. These results imply that accelerated fatigue development in *Tfam* KO muscle is due to impaired mitochondrial oxidative capacity rather than increased energy cost of contraction, but further experiments directly addressing this issue are needed to provide a solid answer to this question.

The post-stimulation recovery kinetics of phosphocreatine can be used as an index of the mitochondrial oxidative capacity *in vivo* (Arnold et al., 1984; Lanza et al., 2011). The rate of phosphocreatine recovery was markedly decreased and the 10-min recovery period was not long enough for a complete recovery in *Tfam* KO mice, which is in agreement with the slow phosphocreatine recovery kinetics commonly reported in patients with mitochondrial myopathies (Barbiroli et al., 1997; Bendahan et al., 1992; Kornblum et al., 2005; Moller et al., 2005; Radda et al., 1982; Taylor et al., 1994). Intriguingly, the slowing of phosphocreatine recovery was similar in 11- and 14-week-old *Tfam* KO mice, thereby suggesting that the decline in mitochondrial oxidative capacity was not progressing within this time frame. This is in contrast to the faster initial decrease in phosphocreatine during the induction of fatigue at 14 weeks of age than at 11 weeks of age. Lower phosphocreatine in muscle of exercising humans has been linked to reduced oxygen supply (Haseler et al., 2004) and slower pulmonary O_2_ uptake, and heart rate kinetics at the onset of moderate-intensity exercise has been observed in mitochondrial myopathy patients (Grassi et al., 2009). Thus, the faster initial rate of phosphocreatine consumption during exercise in the *Tfam* KO mice at 14 weeks of age, as well as in mitochondrial myopathy patients, appears to involve factors other than the decrease in mitochondrial oxidative capacity in skeletal muscle.

Excessive Ca^2+^ accumulation in mitochondria during contraction has been suggested to be a main causative factor of muscle weakness in *Tfam* KO mice (Aydin et al., 2009), and the SR-mitochondria interface has been proposed to be the Ca^2+^ entry site (Gineste et al., 2015). Indeed, MAM can allow Ca^2+^ fluxes from the SR to mitochondria and thereby speed up mitochondrial respiration (Csordas et al., 2018). The present results show similar amounts of MAM in *Tfam* KO and wild-type muscle, whereas the number of contact points between VDAC in the outer mitochondrial membrane and the SR Ca^2+^ channel IP_3_R1 was lower in *Tfam* KO muscle. Obviously, the reduced number of VDAC-IP_3_R1 contact points cannot explain the excessive mitochondrial Ca^2+^ uptake in *Tfam* KO muscle. Importantly, the aberrant mitochondrial Ca^2+^ accumulation in *Tfam* KO muscle fibers occurs during repeated contractions when SR Ca^2+^ release is mediated via another Ca^2+^ channel, ryanodine receptor 1 (Hernandez-Ochoa and Schneider, 2018; Santulli et al., 2017; Shishmarev, 2020). Furthermore, on the mitochondrial side, the MAM-related matrix protein cyclophilin D (also called peptidyl-prolyl cis-trans isomerase F) is involved in excessive Ca^2+^ uptake, as illustrated by results showing (1) partial inhibition of the uptake by application of the cyclophilin D-binding inhibitor cyclosporine A; (2) increased protein expression of cyclophilin D in skeletal muscle from both *Tfam* KO mice and mitochondrial myopathy patients; and (3) an increased lifespan of cyclosporine A-treated *Tfam* KO mice (Aydin et al., 2009; Gineste et al., 2015). Thus, the contraction-mediated aberrant mitochondrial Ca^2+^ accumulation in *Tfam* KO muscle is unlikely to occur via VDAC-IP_3_R1-dependent interactions, but a role for MAM that involves ryanodine receptor 1-cyclophilin D interactions cannot be excluded.

Although the present *Tfam* KO mouse model mimics major features of human mitochondrial myopathies, it also has limitations. For instance, *Tfam* is knocked out only in fast-twitch muscle fibers so that there are still intact slow-twitch fibers that may disguise functional impairments, unlike patients with mitochondrial myopathy, in which mutations affect all muscle fiber types. Moreover, mitochondrial mutations in humans will be present in various cell types, and functional impairments can be seen in organs other than skeletal muscle. Finally, *Tfam* KO mice show a rapidly progressing myopathy with only a few weeks between the first overt symptoms and death, which makes investigations in the later stage of the disease cumbersome.

In conclusion, disorders related to an impaired mitochondrial function are highly diverse and complex with different organs being affected in a rather unpredictable manner. Numerous mouse models have been generated to better understand the pathogenic mechanisms underlying mitochondrial diseases (Ishikawa and Nakada, 2021). In this study, we used fast-twitch muscle fiber-specific *Tfam* KO mice and showed that this mouse model presents a disease progression similar to that observed in mitochondrial myopathy patients; that is, an initial decline in mitochondrial oxidative capacity accompanied by a faster force decline during repeated contractions (i.e. accelerated development of muscle fatigue), which is followed by a progressive decrease in force production in the unfatigued state (i.e. muscle weakness), reduction of bodyweight and premature death. Thus, the present *Tfam* KO mice provide a good model for studies of mechanisms underlying muscle defects at different stages of mitochondrial myopathies, and the knowledge gained can be used to propose novel treatment strategies for these serious diseases for which no effective treatment currently exists.

## MATERIALS AND METHODS

### Animals

*Tfam* KO mice and their wild-type littermates were used for experiments. The fast-twitch skeletal muscle fiber-specific *Tfam* KO mouse model was generated as described previously (Wredenberg et al., 2002). A first group of mice was tested at 11 weeks of age (i.e. before mice enter the stage with severe phenotype) and a second group at 14 weeks of age (i.e. when mice are about to enter the terminal stage with weight loss and a marked reduction in force production) (Wredenberg et al., 2002). All experiments were conducted in agreement with the French guidelines for animal care and in accordance with the European Convention for the Protection of Vertebrate Animals used for Experimental and other Scientific Purposes, and institutional guidelines 86/609/CEE November 24, 1986. All animal experiments were approved by the Institutional Animal Care Committee of Aix-Marseille University (permit number, 12522-2017121119249655 v1). Mice were housed in an environment-controlled facility (12-12 h light-dark cycle at 22°C) and received water and standard food *ad libitum*.

### Study design

Functional, anatomical and metabolic investigations of the left hindlimb muscles of wild-type and *Tfam* KO mice were performed strictly non-invasively *in vivo*. Bodyweight was measured before mice were subjected to the following protocol. The mechanical performance was assessed by measuring the maximal tetanic force of plantar flexor muscles [*gastrocnemius* (Gas), *soleus* (Sol), and *plantaris*], and force production of plantar flexor muscles during a fatiguing protocol. Metabolic changes were evaluated before, during and after the fatiguing stimulation protocol using ^31^P-MRS. Before the fatiguing protocol, the volume of hindlimb muscles was quantified by MRI. Mice were euthanized by cervical dislocation at the end of the force and magnetic resonance experiments.

### Animal preparation

Mice were initially anesthetized in an induction chamber (Equipement vétérinaire; Minerve, Esternay, France) with 4% isoflurane in 33% O_2_ (0.5 l/min) and 66% N_2_O (1 l/min), and placed supine in a cradle designed in our laboratory. A custom-built facemask continuously supplied with 1.75% isoflurane in 33% O_2_ (0.2 l/min) and 66% N_2_O (0.4 l/min) was incorporated into the cradle, and was used to maintain a prolonged anesthesia throughout the experiment. The hindlimb was centered inside a 16-mm-diameter ^1^H Helmholtz imaging coil and the belly of the Gas muscle was located above an elliptical (6×8 mm) ^31^P-MRS surface coil. The foot was positioned on the pedal of a custom-made ergometer with a 90° flexion ankle joint. A force transducer was part of the pedal as described previously (Giannesini et al., 2010). Two rod-shaped surface electrodes integrated into the cradle and connected to an electrical stimulator (Digitimer, model DS7A, Welwyn Springs, UK) were placed on the left hindlimb, one at the heel level and the other one just above the knee joint.

### Force output measurements

The analog electrical signal coming out from the force transducer was amplified with a home-built amplifier (Operational amplifier AD620; Analog Devices, Norwood, MA, USA), converted to a digital signal, monitored and recorded on a personal computer using the Powerlab 35/series system (AD Instruments, Oxford, UK). Skeletal muscle contractions were achieved by non-invasive transcutaneous electrical stimulation elicited with square-wave pulses (0.5 ms duration). The individual maximal stimulation intensity was determined by progressively increasing the stimulus intensity until there was no further increase in peak twitch force. Maximal tetanic force of the plantar flexor muscles was assessed in response to a 150-Hz pulse train (duration, 0.75 s) and submaximal force in response to 20 Hz (duration, 0.75 s). Force production of plantar flexor muscles was also measured during a fatigue protocol (80 contractions, 40 Hz, 1.5 s on, 6 s off).

The peak force of each contraction was measured using LabChart software (AD Instruments). For the fatigue protocol, the tetanic force was averaged every 5 contractions for the sake of clarity. The total force production was computed as the sum of each individual peak force measured during the fatigue protocol. The C_50_ corresponded to the contraction number at which the force amplitude was 50% of the initial force of the fatiguing exercise. Force was normalized with respect to the volume of hindlimb muscles (see below) to obtain specific force (in mN/mm^3^).

### Magnetic resonance experiments

Investigations were performed using a 47/30 Biospec Avance MR system (Bruker, Karlsruhe, Germany) equipped with a 120-mm BGA12SL (200 mT/m) gradient insert.

#### Anatomical imaging

MRI data were acquired at rest, i.e. before the fatiguing protocol. Fifteen consecutive contiguous axial slices (thickness, 0.7 mm), covering the region from the knee to the ankle, were selected across the lower hindlimb. Rapid acquisition with relaxation enhancement (RARE) images (RARE factor, 4; effective echo time, 22.5 ms; actual echo time, 10.6 ms; repetition time, 1000 ms; number of averages, 10; number of repetitions, 1; pulse type, hermite; pulse duration, 2 ms; flip angle, 90°; sweep width, 2.7 kHz; field of view, 4.2×4.2 cm; matrix size, 256×192; acquisition time, 8 min 00 s) were recorded.

Images were analyzed using FSLview (Functional MRI of the Brain, Oxford, UK) (Jenkinson et al., 2012). The border of the whole hindlimb muscle area was manually delineated in the two slices located on the proximal and distal parts. Segmentation of the missing intermediate slices was automatically performed on the basis of image registration procedures as described previously (Ogier et al., 2017). The volume of the hindlimb muscles (mm^3^) was calculated as the sum of the volume of the ten consecutive largest slices, which were located on the proximal part and covered the largest volume of the hindlimb muscle belly.

#### Metabolism

^31^P spectra (8-kHz sweep width; 2048 data points) from the posterior hindlimb muscles region were acquired continuously throughout the standardized rest-fatigue-recovery protocol. A total of 800 partially saturated (repetition time=2 s) free induction decays (FID) were recorded.

^31^P-MRS data were processed using a proprietary software developed using IDL (Interactive Data Language, Research System, Inc., Boulder, CO, USA) (Le Fur et al., 2010). The first 180 FID were acquired at rest and summed together (*n*=1; time resolution, 6 min). The next 320 FID were acquired during the stimulation period and summed by blocks of 80 during the stimulation procedure (*n*=4; time resolution, 160 s). The last 300 FID were acquired during the recovery period and summed by blocks of 50 (*n*=6; time resolution, 100 s). The relative concentrations of high-energy phosphate metabolites (phosphocreatine, inorganic phosphate and ATP) were obtained by a time-domain fitting routine using the AMARES-MRUI Fortran code and appropriate prior knowledge of the ATP multiplets. As previously suggested (Chance et al., 1981), inorganic phosphate values were normalized to the sum of phosphocreatine and inorganic phosphate values, which were considered as constant in order to take into account potential movement artifacts. Intracellular pH (pH_i_) was calculated from the chemical shift of the inorganic phosphate signal relative to phosphocreatine (Moon and Richards, 1973). The resting phosphocreatine to ATP ratio was calculated from the peak areas of the phosphocreatine and β-ATP of the spectrum acquired at rest. The initial rate of phosphocreatine degradation (rate of phosphocreatine degradation at the start of exercise; Vi_Phosphocreatine degradation_) was calculated as:
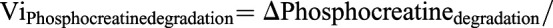

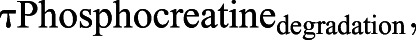
where ΔPhosphocreatine_degradation_ is the percentage of phosphocreatine depletion measured at the exercise end (relative to basal value) and τPhosphocreatine_degradation_ is the time constant of phosphocreatine degradation, which was determined by fitting the time course of phosphocreatine level to a mono-exponential function with a least-mean-squared algorithm. Similarly, the phosphocreatine recovery kinetic parameters [initial rate of phosphocreatine resynthesis at the start of the recovery period (Vi_Phosphocreatine resynthesis_)] and phosphocreatine recovery time constant (τ_Phosphocreatine_) were determined during the post exercise period by fitting the time course of phosphocreatine resynthesis to a mono-exponential function.

### Isolation of mitochondria-associated membranes

Mice were euthanized by cervical dislocation and Gas muscles were extracted and immediately placed in isolation buffer IB1 [225 mM mannitol, 75 mM sucrose, 0.5% bovine serum albumin, 0.5 mM EGTA and 30 mM Tris HCl (pH 7.4)] as described previously (Wieckowski et al., 2009). The tissues were finely minced with scissors and the pieces were then homogenized by applying 10-20 strokes in a glass-Teflon tissue grinder. The homogenates were centrifuged at 740 ***g*** for 5 min, and the supernatants were taken and similarly centrifuged. The new supernatants were collected and centrifuged at 9000 ***g*** for 10 min at 4°C. The pellets were gently resuspended in stop buffer SB [225 mM mannitol, 75 mM sucrose and 30 mM Tris-HCl (pH 7.4)] and centrifuged at 10,000 ***g*** for 10 min. The pellets of crude mitochondria were resuspended in mitochondria resuspending buffer [MRB; 250 mM mannitol, 5 mM HEPES (pH 7.4) and 0.5 mM EGTA]. For purifying mitochondria and MAM, the crude mitochondrial fractions were layered on top of Percoll medium [225 mM mannitol, 25 mM HEPES, 1 mM EGTA and 30% Percoll (v/v)] in 14-ml thin-wall Beckman ultracentrifugation tubes. MRB was added on top of the mitochondrial suspensions to fill up the tubes. The latter were centrifuged at 95,000 ***g*** for 30 min at 4°C. The MAM, identified as a round white layer in the middle of the tube, were collected and washed by MRB through centrifugation at 6300 ***g***/10 min/4°C and then recentrifuged in polycarbonate tubes using a 100,000 ***g***/1 h/4°C ultracentrifugation. The ring of MAM at the bottom of the tubes was finally collected. Protein concentration was quantified using the Lowry method.

### *In situ* proximity ligation assay

VDAC1 (ab14734) and IP_3_R1 (sc28614) proximity were measured using an *in situ* proximity ligation assay [PLA; Sigma-Aldrich Duolink anti-rabbit plus (DUO92002) and anti-mouse minus (DUO92004) kits] to detect and quantify SR-mitochondria interactions, as described and thoroughly validated previously (Tubbs and Rieusset, 2016; Tubbs et al., 2014). In muscle tissue, *in situ* PLA were performed on paraffin-embedded sections, after an antigen retrieval at pH 6, using a bright-field revelation. Dots were quantified using Image J. Results were expressed as number of dots per field. A minimum of ten images per muscle were analyzed.

### Statistical analysis

Data are presented as mean±s.e.m. Statistical analyses were performed using GraphPad Prism software (version 7.04). Normality was checked using a Shapiro–Wilk test. Parametric tests were performed when data were normally distributed. If they were not normally distributed, non-parametric tests were used. Student's unpaired *t*-test or an unpaired Mann–Whitney test was used to compare VDAC-IP3R1 interaction and MAM, respectively. Two-way ANOVA (genotype×age) was used to compare WT-11w, *Tfam* KO-11w mice, WT-14w and *Tfam* KO-14w mice. Two-way ANOVA with repeated measures on time or contraction number was used to compare force production or metabolic changes during induction of fatigue and recovery in the four groups. When a significant interaction was found, Tukey's post-hoc test was performed. When a main effect was found, Sidak's post-hoc test was performed. Statistical significance was accepted when *P<*0.05. For the sake of clarity, when an interaction was found, we only show significant differences between wild-type and *Tfam* KO mice at either 11 or 14 weeks of age, or 11 weeks and 14 weeks of age for either wild-type or *Tfam* KO mice (i.e. statistical differences between wild type at 11 weeks versus *Tfam* KO at 14 weeks, and wild type at 14 weeks versus *Tfam* KO at 11 weeks are not shown).

## Supplementary Material

Supplementary information
