# Supplementary material for: Impaired aerobic capacity and premature fatigue preceding muscle weakness in the skeletal muscle Tfam-knockout mouse model
Source: Dis Model Mech. 2021 Sep 15;14(9):dmm048981. doi: 10.1242/dmm.048981 (PMC8461820; doi:10.1242/dmm.048981)
Supplement: Supplementary information [file dmm-14-048981-s1.pdf]

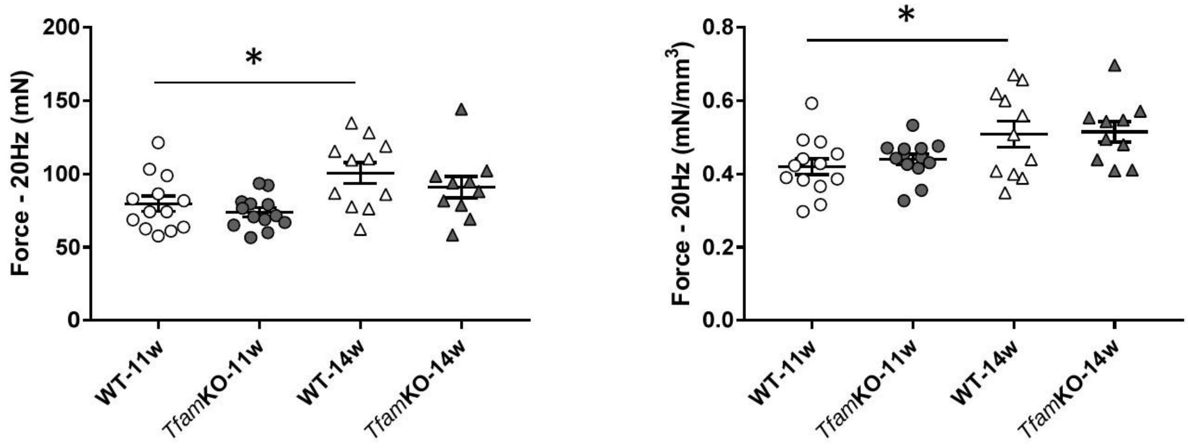

Fig. S1. Absolute and specific submaximal forces in the unfatigued state were unaltered in *Tfam* KO mice. Absolute (left panels) and specific (right panels) force production in response to 20 Hz stimulation. WT-11w, *n*=13; *Tfam* KO-11w, *n*=13; WT-14w, *n*=11; *Tfam* KO-14w, *n*=10. Data presented as individual values and mean±s.e.m. Significant difference **P*<0.05 (two-way ANOVA with Sidak's post hoc test).

Disease Models & Mechanisms • Supplementary information

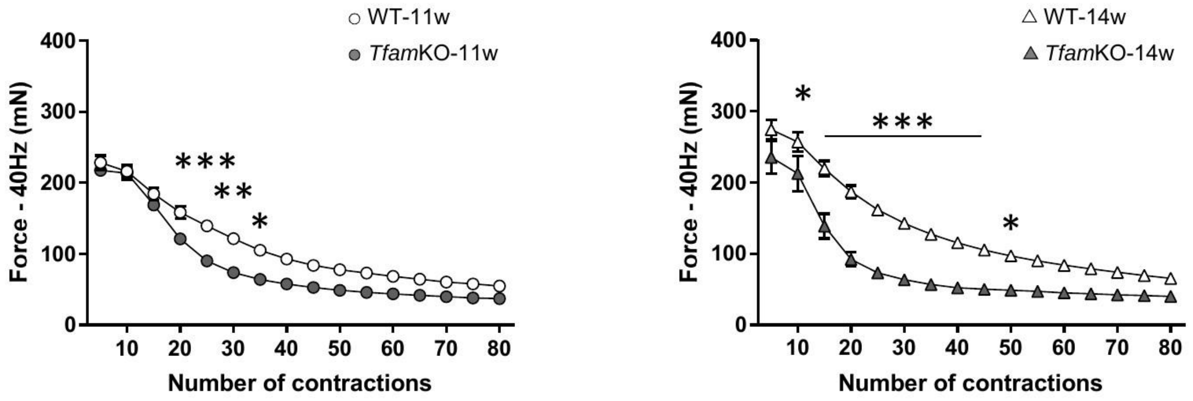

Fig. S2. Absolute force decreased more rapidly during exercise in *Tfam* KO mice. *In vivo* absolute force of the plantar flexor muscles during the fatiguing stimulation protocol at 11 weeks (left panel) and 14 weeks (right panel) in WT and *Tfam* KO mice. WT-11w, *n*=13; *Tfam* KO-11w, *n*=13; WT-14w, *n*=11; *Tfam* KO-14w, *n*=10. Values are mean±s.e.m. Significant difference **P*<0.05, ** *P*<0.01 and *** *P*<0.001 (two-way ANOVA with repeated measures on contraction number and Tukey's post hoc test).

Disease Models & Mechanisms • Supplementary information

Table S1. Metabolites at rest and at the end of the 10 min recovery period post-exercise in WT and *Tfam* KO mice.

Metabolites	11 weeks		14 weeks	
	**WT**	***Tfam* KO**	**WT**	***Tfam* KO**
$[PCr]_{rest}$ (%)	100	100	100	100
$[PCr]_{end\ recovery}$ (%)	93±2	81±2*** ##	95±2	78±5*** ###
$Pi/(PCr+Pi)_{rest}$	0.07±0.01	0.17±0.01	0.08±0.01	0.25±0.04
$Pi/(PCr+Pi)_{end\ recovery}$	0.14±0.02	0.32±0.02*** ### $	0.12±0.02	0.42±0.04*** ### $$

WT-11w, n=13; *Tfam* KO-11w, *n*=13; WT-14w, *n*=11; *Tfam* KO-14w, *n*=9. Values are mean±SEM. Significantly different from rest (same age and same genotype) **P*<0.05 and ****P*<0.001. Significant difference between genotypes (same age and same condition) ##*P*<0.01 and ###*P*<0.001. Significant difference between ages (same genotype and same condition) $*P*<0.05. One-way ANOVA and Tukey's post hoc test for all comparisons. PCr, phosphocreatine; Pi, inorganic phosphate.

Disease Models & Mechanisms • Supplementary information
